# Women with first recurrence of endometrial cancer: who are they? An analysis of real-world data of the JAGO/NOGGO

**DOI:** 10.1186/s12885-025-15168-8

**Published:** 2025-12-02

**Authors:** Louisa Proppe, Julia Sarah Maria Zimmermann, Jens Hachenberg, Teresa Pan, Irina Tsibulak, Julia Caroline Radosa, Christine Botscharnikow, Chiara Flethe, Jalid Sehouli, Klaus Pietzner

**Affiliations:** 1 Young Academy of Gynecologic Oncology (JAGO), North-Eastern German Society of Gynecologic Oncology (NOGGO), Berlin, 13359 Germany; 2 Department of Gynecology and Obstetrics, University Medical Center Schleswig-Holstein, Campus-Lübeck, Lübeck, Germany; 3 Department of Gynecology and Obstetrics, University Hospital Münster, Münster, Germany; 4 Department of Gynecology, Obstetrics and Reproductive Medicine, Saarland University Hospital, Homburg, Germany; 5 Department of Gynecology and Obstetrics,, Hannover Medical School, Hannover, Germany; 6 Department of Obstetrics and Gynecology, , Medical University of Innsbruck, Innsbruck, Austria; 7 Department of Gynecology, Center for Oncological Surgery, Charité-Universitätsmedizin Berlin, Corporate Member of Freie Universität Berlin, Humboldt- Universität Zu Berlin, Berlin Institute of Health, Berlin, Germany

**Keywords:** Endometrial cancer recurrence, Immune checkpoint inhibition, Chemotherapy, Cancer treatment

## Abstract

**Background:**

Recurrent endometrial cancer is still associated with poor survival rates. Daily life factors and comorbidities influence adherence to oncologic treatment. This study provides multicenter real-world data on patients life conditions and tumor’s characteristics, which might contribute to treatment decision-making.

**Materials and methods:**

The study was performed retrospectively in five centers for gynecologic oncology. All patients treated for recurrent endometrial cancer between 2005 and 2022 were included, regardless of whether they received curative or palliative, surgical or medical treatment. Data collection was performed using the RedCap^®^ software (Research Electronic Data Capture).

**Results:**

In total, 277 patients with recurrent endometrial cancer were included in the study. The median age at time of recurrence was 70 years. 68.3 % of the patients had distant metastases, most of them (41 %) had pulmonary metastases. 63.9% of the patients had pelvic recurrences. 30.7 % of the patients had a second recurrence and 49.6 % of all patients died during the follow-up. 86.3 % of patients died due to endometrial cancer. Polypharmacy as an indicator of frailty was analyzed revealing that 34.2 % of the patients took more than five different drugs per day.

**Conclusion:**

The findings of this study indicate that the majority of patients with recurrent endometrial cancer undergo tumor-specific treatment rather than best supportive care. However, in cases of recurrence, the carcinoma itself remains a very frequent cause of death. Considering that a significant number of patients are living independently at the time of diagnosis, this study may contribute to treatment plans that prioritize strategies that enable patients to stay at home and maintain their autonomy.

**Key message:**

The present study suggests that most patients with recurrent endometrial cancer are in relatively good overall health, allowing them to undergo anti-tumor treatment. In general, they live independently and prefer active treatment over best supportive care.

## Background

Even though endometrial cancer is often curable, a significant proportion of women still experience relapse. Recently, treatment for endometrial cancer has evolved significantly. The introduction of molecular subgroups, as outlined in the latest European Society of Gynecologic Oncology (ESGO) guidelines, has brought major changes to risk classification and treatment decisions at the time of initial diagnosis [1]. Despite new treatment options, recurrent endometrial cancer remains associated with poor survival rates [2]. It was not until 2016 that the publication of the ESMO-ESGO-ESTRO Consensus Conference by N. Colombo et al. established a standard of care for treating recurrent endometrial cancer. Just a few years later, the integration of molecular subgroups into clinical practice led to significant changes in treatment approaches [3].

The risk of recurrence in endometrial cancer patients varies between 3 and 40%, depending on factors such as tumor stage, cancer subtype, oncologic therapy, and partially unknown risk factors [4, 5]. Regardless of the molecular subgroup, tumor size is a major risk factor. Tumors larger than 20–25 mm are carrying a higher risk of recurrence compared to those smaller than 20 mm [6, 7].

Retrospective data published in 2020 showed that 40.3% of high-risk patients experienced any recurrence, whereas only 17% of the low and intermediate-risk group had a recurrence. Among 64% of the recurrent cases, metastasized at distant locations [4]. Nearly 90% of the recurrences occur during the first three years after the first diagnosis [8]. The 3-year overall survival rates vary significantly with 73% survival in cases of local recurrences, 14% in cases of pelvic recurrences, and only 8% in patients with distant metastases [9].

However, the heterogeneity within the patient population can complicate clinicians’ treatment decisions. In daily practice, it is often assumed that patients with recurrent endometrial cancer may be frail. Frailty is a well-known predictive factor for surgical morbidity [10] However, data on the clinical condition of patients with recurrent endometrial cancer remain limited. Since every treatment carries a risk of morbidity, and endometrial cancer management has undergone significant changes in a short period, it is essential to assess our patients and their medical condition. Additionally, social circumstances play a crucial role in oncologic outcomes and are rarely analyzed in literature [11].

Until now, most knowledge about these newly approved treatment options has been derived from clinical trials. Real-world data, as analyzed in this study, can provide valuable insights into how these treatments can be effectively integrated into daily patient care.

Life-conditions primarily refer to how patients are managing their daily life, including the presence of comorbidities and concurrent medications. Clinical parameters include age, BMI, endometrial cancer type (including molecular subgroup, if available), and localization of recurrence, based on findings in the patients records. This knowledge may help guide treatment decisions, preserving quality of life, minimizing therapy-related toxicity while optimizing oncologic outcomes. The objective of this study is to provide real-world evidence on the quality of life of patients with recurrent endometrial cancer, an area that is largely underreported in scientific literature.

## Materials and methods

The study was performed retrospectively in five centers for gynecologic oncology (University Medical Center Schleswig-Holstein, Campus Lübeck, Germany; Saarland University Hospital (Homburg), Germany; University Medical Center Hannover, Germany; Charité Medical University, Berlin, Germany; and Medical University of Innsbruck, Austria). Data were analyzed after being transferred to an anonymized service database. In accordance with the journal’s guidelines, we will provide our data for independent analysis by a selected team by the Editorial Team for the purposes of additional data analysis or for the reproducibility of this study in other centers if such is requested.

Patients treated for recurrent endometrial cancer between 2005 and 2022 were included in the study. All patients were included, regardless of whether they received curative or palliative, surgical or medical treatment. Patients for whom the exact timing of recurrence diagnosis and/or initial diagnosis and information on histological subtype were unavailable were excluded. To ensure reproducibility of the analysis, patients with incomplete data were excluded, as timelines could not be calculated. There were no further exclusion criteria. Only for the visualization of recurrence patterns, we categorized different recurrence types: pelvic recurrence was defined as any recurrence within the pelvis, including local lymph node involvement, vaginal recurrence, or other pelvic recurrences, involvement of other organs was considered as distant metastases.

Data collection on clinical and pathologic factors was performed using the RedCap^®^ software [12]. The 2009 FIGO (Féderation Internationale de Gynecologie et d’Obstetrique) classification for endometrial cancer has been used. Body mass index (BMI) was indicated in kg/m^2^. Obesity was defined as a BMI ≥ 30.0 kg/m^2^.

Molecular profiling for p53, ER, PR, and MMR-deficiency has been performed using immunohistochemical analysis as part of clinical routine. POLE-mutations were analyzed with sequencing methods. L0 was obtained from pathology results and defined as no lymph node invasion of tumor cells, while L1 was defined as tumor cells in lymphatic vessels of the tumor region. LVSI, adopted by pathology results, included tumor invasion in both lymph and blood vessels and was defined as the presence of tumor cells in a space lined by endothelial cells outside the immediate invasive border. In accordance with ESGO guidelines, LVSI positivity was defined by the pathological report as multifocal or diffuse infiltration of tumor cells involving five or more lymphovascular spaces [1].

Statistical analyses were performed with the SPSS Statistics software (version 27; IBM Corporation, Armonk, NY, United States of America). Qualitative and quantitative data are presented as absolute and relative frequencies as well as medians and range, respectively.

### Ethical approvement

This study is performed according to the declaration of Helsinki. The ethics committees in charge approved conduction of the study with no adjustments (ethic approval No 22–225, 10578_BO_K, _2022, 144/22, 1237/2022).

## Results

A total of 277 patients with sufficient documentation were included in the study. Median age at time of diagnosis was 68 (range 37–92) years, median age at time of recurrence was 70 (range 37–93) years. After the initial diagnosis of endometrial cancer, patients were diagnosed with their first recurrence after a median time of 16 months. The median BMI accounted for 29.1 kg/m^2^ at time of diagnosis and for 28.2 kg/m^2^ at time of recurrence. Median number of deliveries was 1.8 (0–12) (Table 1).

At initial diagnosis, 200 patients (76%) were treated at a university hospital, while at time of recurrence 232 (88.2%) patients were treated there. The local gynecologist was the referring physician for further treatment in 124 (73.4%) of all patients at time of recurrence (Table 1).

**Table 1:** .

Characteristic		*N* (%) = 277
Age at initial diagnosis (median, min-max; years)		68 (range 37–92) years
Age at recurrence (median, min-max; years)		70 (range 37–93) years
BMI at initial diagnosis (median; kg/m ^2^)		29.1 kg/m2
BMI at recurrence (median; kg/m^2^)		28.2 kg/m2
Type of hospital treatment at initial diagnosis		
University hospital		200 (76%)
Tertiary hospital with gynecologic oncology department		33 (12.5%)
Tertiary hospital without gynecologic oncology department		30 (11.4%)
Type of hospital treatment at recurrence		
University hospital		232 (88.2%)
Tertiary hospital with gynecologic oncology department		19 (7.2%)
Tertiary hospital without gynecologic oncology department		12 (4.6%)
FIGO stage	I	126 (48.8%)
	II	34 (13.2%)
	III	72 (27.9%)
	IV	26 (10.1%)
Grading	1	55 (21.7%)
	2	92 (36.4%)
	3	106 (41.9%)
Histology at time of diagnosis	endometrioid carcinoma	191 (74.6%)
	serous carcinoma	37 (14.5%)
	clear cell carcinoma	10 (3.9%)
	undifferentiated carcinoma	18 (7%)
Histology at time of recurrence	endometrioid carcinoma	140 (70%)
	serous carcinoma	32 (16%)
	clear cell carcinoma	10 (5%)
	undifferentiated carcinoma	18 (9%)
Lymphovascular space invasion	L0	151 (70.9%)
	L1	62 (29.1%)

### Social status, comorbidities and co-medication

The social status and comorbidities play a crucial role in the disease and treatment process. For detailed information on comorbidities and comedication, see Table 2. While the majority of patients had secondary diagnoses, 18.4% did not have any other diseases besides endometrial cancer. For those who were on medication, 65.7% took between one to five different medications. Notably, 38.1% of patients regularly took analgesic agents. At the time of recurrence diagnosis, 22.2% of patients did not take any medication, and only 12% of the patients were using metformin as a co-medication. However, 8.8% of patients were taking more than ten different medications per day.

**Table 2:** .

Comorbidity	*N* (%) = 277
Obesity	90 (32.5%)
Arterial hypertension	162 (58.5%)
Diabetes	54 (19.5%)
Cardiac insufficiency	18 (6.5%)
COPD	6 (2.2%)
Comedication	
Metformin	30 (12.2%)
Beta blockers	61 (40.4%)
Pain medication	56 (38.1%)
Diuretics	55 (36.7%)
ACE inhibitors	69 (46.3%)
Psychiatric drugs	35 (23.6%)
Anticoagulation	63 (40.1%)
Alcohol consumption	
Not drinking alcohol	125 (80.1%)
Drinking alcohol occasionally	24 (15.4%)
Drinking alcohol regularly	7 (4.5%)
Social status	
Married	104 (55.6%)
Living with a partner	8 (4.3%)
Living alone	75 (40.1%)
ASA score	
ASA I	4 (1.9%)
ASA II	106 (49.5%)
ASA III	97 (45.3%)
ASA IV	7 (3.3%)

Most of the patients (88.8%) lived independently, sometimes with support of their families.

With respect to anesthesiologic risk, 45.3% of patients were classified as ASA III (American Society of Anesthesiologists), indicating elevated perioperative risk; approximately half were ASA II, according to the patients records (Table 1). Additionally, 34.2% of the patients were taking more than five different drugs a day, and 8.8% were taking even more than ten different medications.

### Characterization of the tumor at time of initial diagnosis

At time of initial diagnosis 191 women (74.6%) had endometrioid tumors, 37 (14.5%) had serous tumors, 10 patients (3.9%) had clear cell histology and 18 (7%) had tumors without differentiation. 126 (48.8%) were in FIGO stage I at time of initial diagnosis and 106 patients (41.9%) suffered from G3 tumors. For detailed tumor characteristics see Table 1.

### Symptoms and treatment of endometrial cancer at initial time of diagnosis

At initial time of diagnosis, 144 patients (52%) presented with vaginal bleeding. All patients (100%) underwent stage-adjusted surgery at time of initial diagnosis. This included either a laparoscopic approach or laparotomy, with at least hysterectomy and adnexectomy, depending on the carcinoma. Resection of other affected organs or structures was performed as needed.

Based on current guidelines and tumor characteristics, systematic lymphadenectomy was performed when indicated. In 152 (58.9%) systematic lymphonodectomy was performed, while in 43 patients (17.8%) sentinel-node biopsy was performed. 224 (90.3%) had R0 resection according to local pathology results.

44 patients (16.7%) patients had external beam radiation therapy; 118 patients (44.9%) had brachytherapy. 99 women (40.9%) had adjuvant chemotherapy, 81 (83.5%) received a chemotherapy consisting of carboplatin AUC 5 q3w and paclitaxel 175 mg/m^2^ q3w, with 75 women (72.8%) receiving three to six cycles of chemotherapy.

### Characterization of the recurrence

**Fig. 1:**
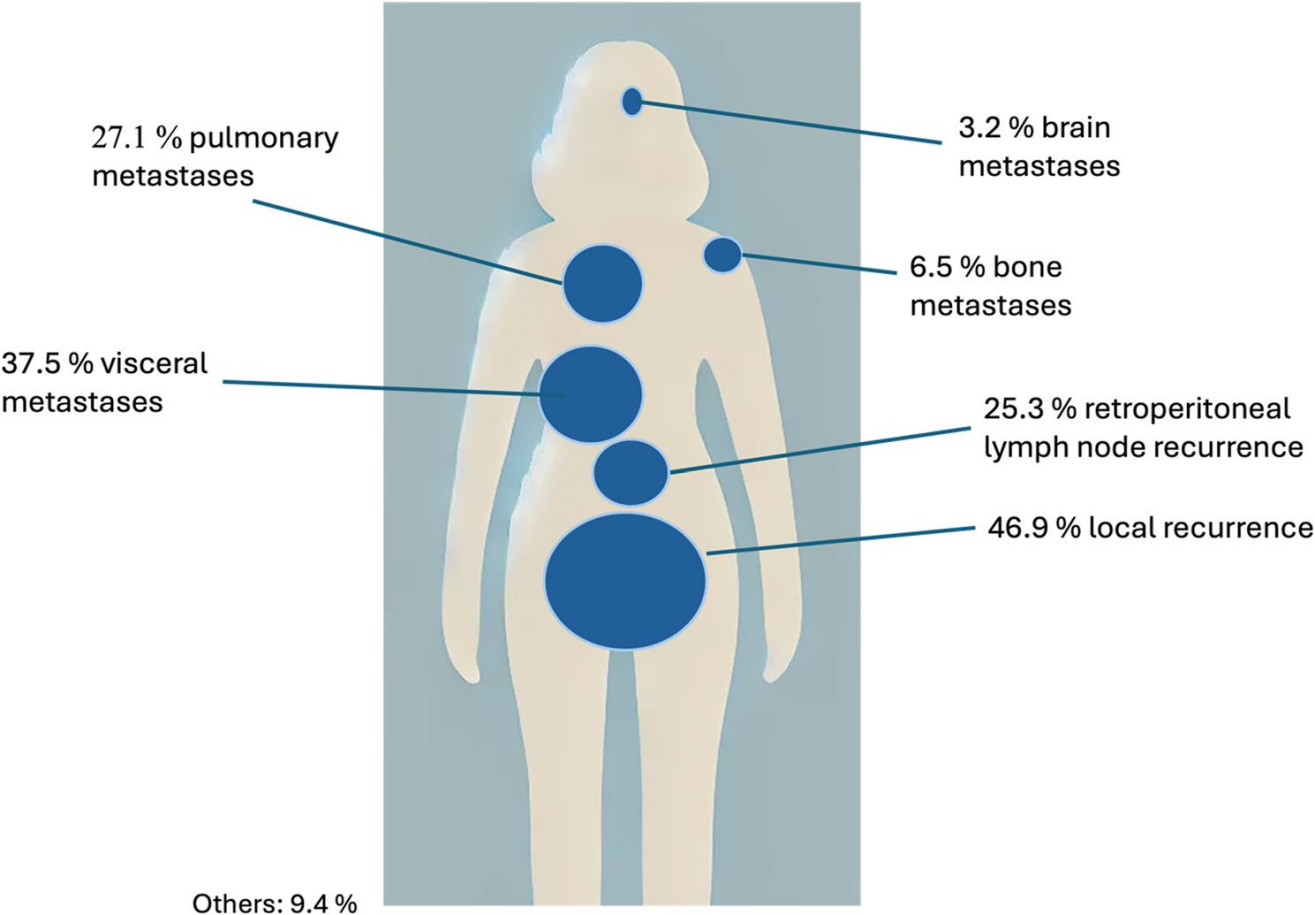
Localization of recurrent endometrial cancers

Recurrence was a local relapse in 130 (48.5%) of all cases and 183 patients (68.3%) had distant metastases (Fig. 1), with 75 patients (41%) having pulmonary metastases. For detailed location of metastases, see Fig. 1. 71 (30.7%) patients relapsed twice. Figure 1 provides an overview of recurrence localizations.

At time of recurrence 140 women (70%) had endometrioid tumors, 32 (16%) had serous tumors, 10 patients (5%) had clear cell histology and 18 (9%) had tumors without differentiation (Table 1).

### Molecular subtypes

There is very limited information available regarding the molecular subgroups, with only 40 patients of the study population having been analyzed. In this small cohort, we did not identify any POLE mutated patients. 8 relapsed patients were p53 aberrant, 17 patients classified to be MMR deficient, and 15 patients were NSMP (no specific molecular profile). Due to the inclusion period (2005–2022) there were information on the molecular subgroup only available for a few patients.

### Symptoms and treatment of recurrence

Only 53 patients (19.1%) presented with vaginal bleeding at the first recurrence, while 106 (38.3%) patients had nonspecific symptoms such as pain, fatigue or weight loss.

**Fig. 2:**
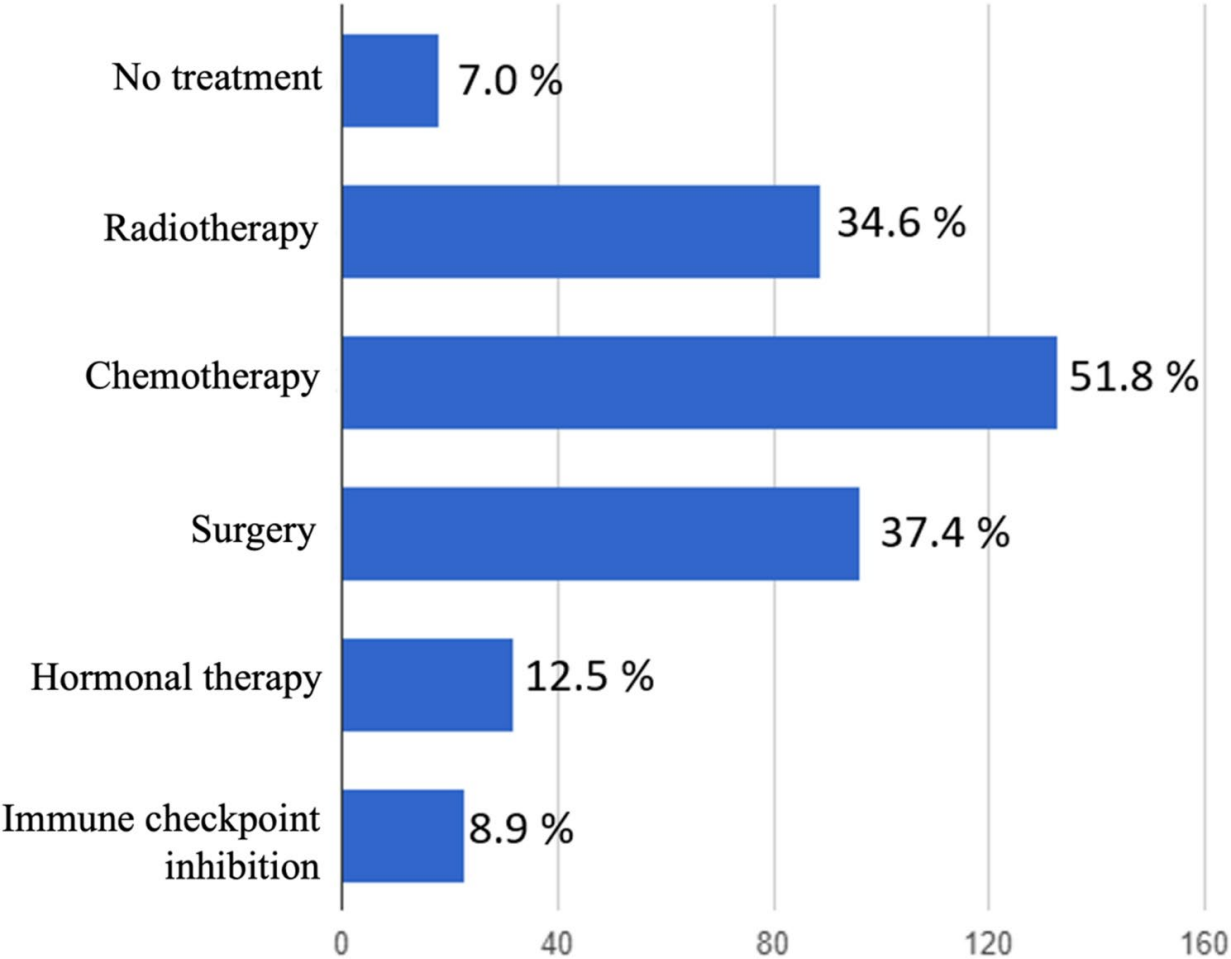
Treatment of recurrent endometrial cancer

In our cohort, the majority of patients received chemotherapy. Therapies might be combined so that 34.7% of the patients underwent stage-adjusted surgical treatment (defined as maximal possible tumor debulking) and a comparable number of patients received either a radiotherapy alone or a postoperative radiotherapy (Fig. 2). 17 patients of the patients undergoing surgery in case of relapse (18.5%) were treated by laparoscopy, while 53 (57.6%) women were treated by laparotomy at time of recurrence. The remaining patients (23.9%) were treated by different surgical approaches, which was either a local resection of the recurrence of a vaginal approach. Of all patients who were treated by surgery, 48 (53.9%) had R0 resection according to local pathology results.

Based on the analysis of patients records, we examined the disease progression after diagnosis of recurrent endometrial cancer. 71 patients (30.7%) experienced a second recurrence, while 160 patients (69.3%) did not. In total, 123 patients (49.7%) died. The cause of death is mostly unknown, but if known, 86.3% of the patients died of endometrial cancer.

**Fig. 3:**
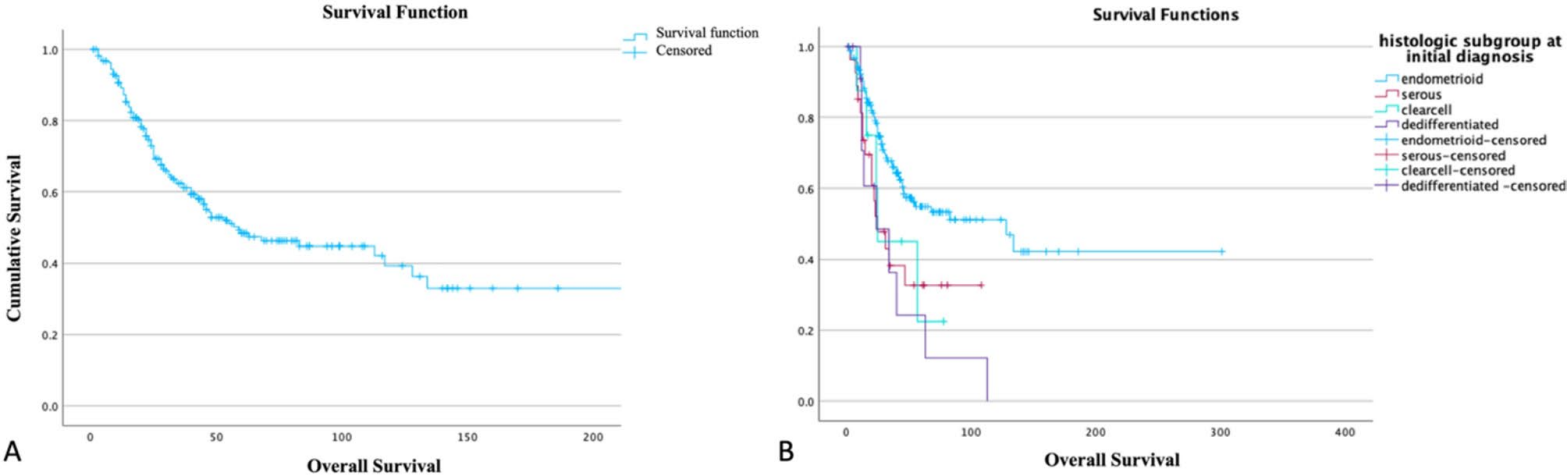
**A** Overall survival of all included patients (median: 59 (31.3–86.7) months; follow-up period up to 200 months). **B** Overall survival dependent on histologic subgroup (OS (median; in months): *p* = 0.002; Endometrioid: 128 (58.2–197.8.2.8); Serous: 25 (11.7–38.3); Clear cell: 25 (22.5–27.5); Dedifferentiated: 24 (0.1–50.5))

Figure 3 shows the overall survival for all patients included in this study. The median overall survival time amounts to 59 (31.3–86.7) months from the time of recurrence. Remarkably, despite all patients experiencing at least one recurrence of endometrial cancer, more than 50% survived for 50 month or more.

## Discussion

In this study, the objective is to analyze patients who experience a recurrence of endometrial cancer. This analysis focuses on living conditions, comorbidities and polypharmacy as potential indicators of frailty. Although these factors strongly impact therapy adherence and treatment outcomes, they remain underrepresented in the published literature.In our cohort, the predominant treatment was chemotherapy, and this pattern might be attributed to the higher incidence of distant metastases rather local recurrences aligning with findings in other publications [4]. Among patients who underwent surgical treatment for recurrence, only 53.9% were treated with R0 resection-. As it is well known that R0 resection is a major prognostic factor in endometrial cancer recurrence, these results are indicating an urgent need of better criteria to identify eligible patients for surgical therapy in recurrent endometrial cancer [13].

We confirm endometrial cancer recurrence remaining a life-threatening condition, as more than 86% of the patients with a known cause of death died from endometrial cancer. However, the mean overall survival of 59 months, despite the high prevalence of distant metastases, is remarkable. In future, this period might be even longer, reflecting the broader use of new treatment options, especially immune checkpoint inhibitors.

As noted by Cashman et al., the median daily drug count for oncologic patients is reported to be seven [14]. Additionally, as analyzed by Woopen et al., polypharmacy plays a pivotal role in the tolerance of chemotherapy among ovarian cancer patients [15]. Their study revealed a significantly higher rate of dose reduction and discontinuation in patients taking five or more medications daily compared to those with fewer medications. In accordance with the literature, our results equally emphasizing the relevance of polypharmacy in this context.

The relatively high number of patients with an elevated anesthesiologic risk (45.3%), indicated by an ASA III score could be attributed to the 37.7% of patients experiencing obesity or potentially be a result of frailty. Self-evidently, compared to the initial diagnosis of the disease, patients tend to be more frail and older when facing a recurrence. Therefore, it is equally crucial to analyze the patients living conditions as it is to examine the tumor itself. Predicting recurrent disease, particularly discerning whether distant metastases or local recurrences may occur, remains challenging [16, 17]. As mentioned earlier, the identification of new endometrial cancer subgroups has only been available for a few years, limiting the current ability to interpret their implication for recurrence patterns.

Heffernan et al. conducted an analysis on 999 patients with recurrent or advanced endometrial cancer, the majority of whom underwent chemotherapy (Carboplatin AUC 5 and Paclitaxel 175 mg/m^2^ q3w) as first and second-line treatments, resulting in an overall survival time of 10.3 months. However, only a small percentage of patients (≤ 3%) received immune-checkpoint inhibition [18]. In our study, the proportion of patients receiving immune-checkpoint inhibition is higher, at 9%. This percentage is increasing, given the approval of the first immune-checkpoint inhibition for recurrent endometrial cancer in Europe was in April 2021 [19]. Since trials such as Keynote-158, the Garnet trial or the Keynote-775, checkpoint inhibition has been implemented to daily clinical routine [20–22]. Due to the retrospective character of the present study dating back to 2005 and the fact that in the early days of checkpoint inhibition approval in recurrent endometrial cancer, its use was clearly not comprehensive from the beginning, we could not report a percentage higher than 9% receiving immunotherapy [18].

The molecular (or immunohistochemical) characterization of endometrial carcinomas, as outlined in the current ESGO guidelines, is relatively recent, resulting in limited information on recurrent endometrial cancer in this retrospective cohort [1]. Subgroup analyses are not feasible due to limited data available on molecular subgroups. Notably, all known subgroups in this study were evenly distributed.

We deliberately chose not to conduct additional subgroup analyses, as this is a retrospective study with inherent limitations.

New treatment options are essential for providing individualized cancer treatment. However, it is important to note that a significant proportion of patients with recurrent endometrial cancer is elderly and frail. This aspect should be carefully considered in treatment planning, alongside the size and localization of the recurrence [23].

In this study, we demonstrated that these considerations align with the real-world scenario, where over one-third of the patients were classified to be ASA III and were taking more than five different medications daily and still have a median survival of 59 months. Confirmations like these, derived from real-world data, play a vital role in validating information obtained from molecular analyzes and clinical trials.

## Conclusion

In summary, our findings indicate that the majority of patients facing a recurrence of endometrial cancer undergo anti-tumor treatment rather than best supportive care. Treatment options vary and should be tailored to individual needs. Polypharmacy is a common factor in endometrial cancer patients and therefor remains an important consideration when treating an elderly population. However, there are no results indicating that polypharmacy is limiting cancer treatment. Notably, in cases of recurrence, the carcinoma itself remains a frequent cause of death. Considering that a significant number of patients are living independently at the time of diagnosis, this study may contribute to treatment plans that prioritize strategies that enable patients to stay at home and maintain their autonomy. We emphasize to combine real-world data with molecular classifications in future studies to personalize treatment for endometrial cancer recurrence.

### Ethical approvement and consent to participate

This study is performed according to the declaration of Helsinki. The ethics committees in charge approved conduction of the study with no adjustments. The leading ethic committee was the ethic committee of the University Hospital Saarland (ethic approval No 22–225, 10578_BO_K, _2022, 144/22, 1237/2022). Informed consent to participate was waived by the ethic committee of the University Hospital Saarland. The remaining ethic committees that were involved was the ethic committee of the University of Luebeck, Germany, the ethic committee of the University Hospital Hannover, Germany and the ethic committee of the University Hospital Innsbruck, Austria Patients were included in this study in an anonymized manner. For this reason, there was, according to the guidelines of the ethic’s committee, no informed consent for participation in this study obtained. All patients were treated at the university hospitals mentioned above.

## Data Availability

The datasets generated and/or analysed during the current study are not publicly available due to data protection but are available from the corresponding author on reasonable request.

## Abbreviations

BMI: Body-mass-index
ER: Estrogen receptor
PR: Progesterone receptor
MMR: Mismatch repair
FIGO: Féderation Internationale de Gynecologie et d’Obstetrique
ASA III score: American Society of Anesthesiologists
NSMP: No specific molecular profile
OS: Overall survival
PFS: Progression-free survival
MSI: Microsatellite instability
